# East Suffolk and Ipswich Hospital Makes Good

**Published:** 1920-01-17

**Authors:** 


### MAINTAINING THE VOLUNTARY SYSTEM.

### East Suffolk and Ipswich Hospital Makes Good.

A strenuous effort is being made by the East Suffolk and Ipswich Hospital to support the voluntary system. The management are evidently of opinion that the first essential in maintaining this principle is to make adequate provision for the wants of a suffering community. They have, therefore, adopted a report and scheme propounded by their Secretary, Mr. Arthur Griffiths, and presented in 1918. This scheme involves an expenditure of £80,000 on extensions, and it is hoped that the sum will be provided as to £50,000 as a war memorial from the Borough of Ipswich and as to the remaining £30,000 from the 245 parishes, in the administrative County of East Suffolk, in part of West Suffolk and in North-East Essex, which form the hospital district. As far as the appeal for the borough money is concerned it may be considered eminently successful, for out of the £50,000 required £45,000 has already been subscribed. The sum to be provided from the county involves much greater difficulty, as the money has to be collected through numerous small centres dotted over the whole area. However, a scheme has been prepared and a sum of £9,000 received as a nucleus. The general appeal to the county will proceed early in the spring.

A grant of £12,500 has been promised out of the distribution of the Red Cross funds towards the purposes of this extension, but has not yet been received. Anticipating the carrying out of this scheme and with a view to putting its house into order, the board of management has elected various members of the visiting staff to fill the new appointments contemplated, and arrangements have been made for carrying on most of the work in the present permanent accommodation, supplemented by the temporary war wards which have not yet been demolished. Hitherto the medical staff of the hospital consisted of medical officers acting both as physicians and surgeons, with two or three separate departments only; but now a clear-cut line of demarcation has been made between all departments, and there have been elected two physicians, two assistant physicians, three general surgeons, two assistant general surgeons, one gynæcologist, one surgeon in charge of ear, throat, and nose department, one surgeon in charge of genito-urinary department, one surgeon in charge of ophthalmic department, one surgeon in charge of orthopædic department, two medical officers in charge of $x$-ray and electro-therapeutic department, one medical officer in charge of venereal diseases department, and three anæsthetists; also three dental surgeons.

The financial position is one which naturally gives the board some anxiety, and with a less energetic or less modern board would, perhaps, cause consternation and a closing of wards. The financial year of the hospital ends on May 31, and the current financial year started with a deficiency of £5,027, the result of the loss occasioned by the treatment of wounded men in hospital.

The annual subscription list last year amounted to £3,571. This was a record sum, and a determined effort is being made to exceed that sum with the current year's total.

The support given by workmen in the various factories and shops was also a record: £3,561, but a system has since been inaugurated by which many of those who previously gave 1d. per week are now giving 2d. per week on a fixed scale, and it was estimated that the income at this rate would have reached over £4,000 during the current financial year. However, the moulders' strike has blasted any such hopes, and the estimate under that head is now being cut down to £2,750. The management is hoping to get further support from the local authorities for the treatment of cases sent in by them, such as school children—operations for tonsils, adenoids, etc.—while it is hoped that some redress may be obtained from the Ministry of Pensions for the daily loss which is now being incurred in the treatment of pensioners. The question has been mooted of launching a scheme for the regular support of agricultural workers, and if this can be brought to successful fruition a large source hitherto untapped will be available.

An enormous amount of work has been done in this hospital during the war in $x$-ray and electro-therapeutics, and with the coming of Peace that work has been expanded. An Orthopædic Department has now been added, and Mr. Richard Charles, F.R.C.S.I., who has been associated with Sir Robert Jones in his orthopædic work, has been appointed surgeon in charge. Much new apparatus has been installed, and a further complete new $x$-ray apparatus of the very latest type, with powerful radiographic coil, is now about to be installed in the electric department.

To enable the Board to bring about the many varied and sweeping changes which have taken place in the personnel, management, and equipment of this hospital, an entire revision of the Rules and Regulations was necessary. Proceeding step by step, the Board of Management has brought about such radical changes in the Statutes of the institution as to enable the necessary Rules and Regulations on modern lines to be brought into operation. It is quite fair to say that, at the present moment, the East Suffolk and Ipswich Hospital is the most democratically governed institution of its kind in this country; and this, no doubt, accounts in some measure for its popularity amongst the workers, and for the support they generously give to it. By the new Rules no one has a prescriptive right to sit on the Board of Management—it is now an entirely elected body, consisting of eighteen governors, a president, two treasurers, four members from among the vice-presidents and life governors, and four members of the medical staff; the Rules also enact that no less than three of the eighteen shall be directly representative of labour or of the Friendly Societies, also, that there shall be representatives of the country districts, who live outside the confines of the Borough, and that one-third of the elected members shall retire each year and shall not be eligible for re-election. Under the old Rules of the institution, some eighty members had a life seat on the managing body, the total board numbering 100. The active management is to be congratulated on its courage in tackling so colossal a subject and in having such a happy issue out of a very difficult position. A number of younger men have been elected on the board, which is now represented by all classes and shades of society and industry, and professions.

Some additional items of expenditure have been incurred by reconstruction after the war. In the early part of the war the children's open-air wards were handed over to the wounded soldiers, and the children accommodated in the nurses' quarters, the cubicles and other partitions in the latter building having been knocked down

**Maintaining the Voluntary System**—(*continued*).

and turned into children's temporary wards. After the war these buildings had to revert to their former use, but instead of continuing the cubicle system a separate bedroom for each nurse has been built, while two large houses in the vicinity have still to be maintained as nurses' homes, and this will be so until the extension under the County Fund is carried out and new permanent buildings have been added to the nurses' home.

The total cost of nurses for this 250-bed hospital is now estimated at £8,656 per annum.

If one thing more than another has been needed in the past to complete the good work of the hospital it has been a convalescent home, or an auxiliary hospital to which patients could be transferred immediately they were fit to be moved as stretcher-cases after operation. Under the will of the late President of the hospital, Dr. J. H. Bartlet, the residue of his large estate comes to the hospital for the express purpose of building, equipping, and maintaining a convalescent home. A site at Felixstowe—one of the most beautiful sites in the kingdom, right on the sea-front and facing south-east—has been acquired, and so soon as the money is available the building will proceed. In the meantime, for the past year, a boarding-house has been hired, equipped, and has been in use as a temporary convalescent home for the women and children, and during the past six months 250 cases have had the benefit of convalescing by the seaside.

However gloomy the financial outlook may appear, the progressive policy of the Board is warranted by past experience. For instance, the following are the increases under the various heads of income as between 1913 and the financial year just ended: Annual subscriptions increased from £2,212 to £3,571; employees' collections, £1,931 to £3,561; congregational collections, £1,158 to £2,226; donations, £743 to £2,920; invested property, £2,382 to £2,734; while a new source of income, that is, the value of gifts in kind, brings in £1,880, and amounts received from local authorities for venereal disease, pensioners, and surgical tuberculosis, amount to £3,575. If this ratio of increases can be maintained it is surely a good augury for the future.

